# Collectanea

**Published:** 1833-02

**Authors:** 


### COLLECTANEA.

**Floriferis ut apes in saltibus omnia libant,**
**Omnia nos, itidem, depascimur aurea dicta.**

### PATHOLOGY.

*Is it possible to give rise to the Matter of Tubercle, at least locally, by direct Inoculation?* I AM acquainted with only one fact that bears on this point; and, although I am aware that little stress can be laid on a single instance, I think it as well to notice it in this place. About twenty years since, while examining some vertebræ containing tubercles, I grazed slightly the forefinger of the left hand, by a stroke of the saw. The scratch was so small that I paid no attention to it; but on the following day it was slightly inflamed, and there gradually formed in it, and almost without pain, a small roundish tumor, apparently confined to the skin, and which, at the end of eight days, was of the size of a large cherrystone. At this period the epidermis cracked, and showed us the small tumor within, which was yellowish and firm, and in every respect like a crude yellow tubercle. I cauterized it with the deliquescent hydrochlorate of antimony, and felt no pain from its operation. At the end of a few minutes, however, after the fluid had penetrated the whole substance of the tumor, I detached it by a gentle pressure. The caustic had softened it, and made it exactly like a soft friable tubercle. The walls of the cavity which had contained this body were of a pearl-gray colour, slightly semitransparent, and without any redness. I applied the caustic afresh to these. The part soon healed, and I have since found no further effects from the accident.—FORBES' *Translation of* LAENNEC *on Diseases of the Chest.*

---

*Frequency of Diarrhœa and Ulceration of the Intestines in Phthisis.* Out of 112 cases of phthisis, diarrhœa was wanting in one only. In some it preceded any sign of phthisis pulmonalis; in about one eighth, it began at the same time as the disease of the lungs, and attended its whole course, from five to twelve months, and even, in some cases, during the greater part of four or five years; in the majority of cases, it began in the latter half of the existence of phthisis; and in one fourth, it appeared only between the fiftieth and twentieth day preceding death. In these cases, which may be called *the diarrhœa of the latter days*, the intestines were found diseased in every case but one; in one half, there was ulceration of the mucous membrane either of the small or large intestines, or both, and in every case but three the ulcers were small and rare; in four fifths of the cases, the mucous membrane of the colon was reduced to a soft pulp, and was almost always more or less red. The diarrhœa was proportioned to the extent of the organic lesion, and was greater when the membrane was softened than when it was simply ulcerated. *The diarrhœa of long standing* was either remittent or continued. In the former kind, which was observed in fifteen cases, the lesions were the same as in the variety just described, being comparatively slight. The diarrhœa continued from forty-eight days to five months, and the remissions lasted eight, ten, fifteen, or twenty days. The continued diarrhœa of long standing lasted from one to twelve months. Out of forty-one cases, the small intestines were ulcerated in thirty-five; the large, in thirty-one; in twelve cases, the small intestines were ulcerated through their whole course, and in thirteen, the ulcers were as large as an inch in diameter; in nineteen cases, there were large ulcerations in the great intestines; in thirty, the mucous membrane of these was of a pulpy softness, and in seventeen, it was red. As a general rule, it was found that, when a diarrhœa had been of long standing and continued, the ulcers were large and numerous. The diarrhœa was found to be as severe when the ulcers were in the small as when they were in the large intestines; but the much greater frequency of softening of the membrane of the latter (which appears to be the consequence of inflammation) proves that the site of this symptom is much more commonly in the large than in the small intestines. In every case where there was much ulceration, the dirrhœa was not only of long duration and continued, but the stools were also frequent, and the loss of strength and the emaciation were always proportioned to the number and frequency of the stools. There appears to be no foundation for the common opinion of diarrhœa alternating with the sweating in phthisis, or the one being supplementary of the other.—*Ibid.*

---

### PRACTICAL MEDICINE.

*Strychnine in Paralysis.* Dr. Turnbull, in his report of the Huddersfield Infirmary, published in the last number of the Edinburgh Medical and Surgical Journal, states that he has given strychnine in many cases of paralysis, but without any benefit when the malady has been of any duration. He adds, very truly, that, when it is exhibited in recent cases, it is difficult to decide whether it has any, and what, share in producing amendment.

---

### PRACTICAL SURGERY.

*A new Method of stopping Hemorrhage from Wounds.* [From a Correspondent.] Hitherto we have only known either mechanical or chemical means for arresting such hemorrhages. It appears, on the contrary, that, by Binelli's method of treatment, which we recommend to the notice of our contemporaries, there is exercised upon injured blood-vessels, and upon the blood issuing from them, a nearly indirect tonic (*unmittelbar dynamischer*) influence; by means of which, hemorrhages are stopped without ligature or compression of any sort, without the use of the known conglutinations, astringents, or caustics, and does not cause any of those changes of the wounded vessel which we generally see arise after the application of the ligature or of powerful chemical caustics. The means discovered by Dr. Fidele Binelli for effecting this purpose is a clear and perfectly transparent water, almost tasteless, having a slightly empyreumatic odour, in which the senses cannot perceive any distinct chemical agent; namely, neither an earth, a salt, an alkali, an acid, nor a metal. The first public trials, to prove the efficacy of this water in arresting hemorrhage, were instituted in Turin, in 1797, by order of the government; the results of which were considered favorable. Soon after, Binelli died, and the secret for making this water also perished. In the years 1829 and 1830, the successors of Binelli affirmed that they had again discovered this secret, and new trials were instituted, at which I was prevented from being present by an accident which occurred to me during my trials in Italy that year.

The information I received respecting the results of those experiments was highly favorable; rather too much so, in order that I should place implicit confidence in them. I returned to Berlin, taking with me a small quantity of the "aqua Binelli," and waited patiently for a suitable opportunity to put it to the test of experience, which was accidentally afforded me in the form of a small unimportant wound, followed by a slight hemorrhage. I was so surprised at the effects of this water, when applied to the wound, that I requested my friend Dr. Karper, an Italian physician, to send me, through the Prussian ambassador residing at Turin, a considerable quantity of the "aqua Binelli," in order that I might be enabled to make further experiments. When I obtained it, I was enabled to make other trials; the results of which are detailed in the following compressed notice.

In the first place, I tried its effects upon wounds of the larger blood-vessels of animals. I exposed various blood-veſsels of young cats: in one, the femoral artery; of another, the same, with its accompanying vein; of another, the carotid artery; and, lastly, the same, with the internal jugular veins. These were cut differently, some longitudinally, some obliquely, some transversely, and others completely across.* The violent hemorrhage hereby induced yielded, without exception, and without any other means being employed, so soon as a charpie or lint, steeped in the "aqua Binelli," was applied and pressed moderately against the wound for five or ten minutes. Encouraged by this success, I selected larger animals for the same experiments, such as dogs and sheep, at which Professor Rudolphi, Dr. Schulz, physician general, Drs. Urebel, Büttnerr, Bahn, and Mr. Halbach, veterinary surgeon in chief, and many others, were present. The results from the latter were as favorable as those from the former: neither injurious inflammation, nor any other prejudicial reaction of the constitution, were remarked; the cicatrization of the wounds proceeded regularly, and all the animals experimented upon perfectly recovered.

Further encouraged, we determined on trying its effects upon man: 1st, (before the assembled class, in the operating theatre,) after amputation of a finger, where the two digital arteries emitted blood very freely. 2d. In a case of a wound of the hand, caused by a cutting instrument, which entered deep between the metatarsal bones of the thumb and indicator finger, and where the hemorrhage could not be arrested by means of compression or the tourniquet, without the fear of causing gangrene. 3dly. After the extirpation of a hardened inguinal gland, from which a parenchymatous hemorrhage ensued. 4thly. After amputation of the thigh, on account of a scrofulous knee-joint: after the operation, the blood issued with great force, on the least relaxation of the tourniquet, from the crural, perforating, and other muscular arteries, also from the vein. In each case the hemorrhage was quickly and permanently arrested by the "aqua Binelli," without any other aid, and did not cause the least pain on its application, nor produced any discoloration on the surface of the wound; neither eschar nor any other local or general disagreeable effect.

The apparent, remarkable, and quite peculiar effect of the water merits all our attention, as an important object of the healing art may thereby be obtained. Our experiments excited a very lively interest, particularly so as we had not hitherto succeeded in our investigations as to the composition of this water. When all my efforts to discover its composition by various chemical reagents were fruitless, I requested experienced analytical chemists to undertake a more searching investigation of it. Drs. Lindes and Runce, professors of chemistry, Mr. Kleist, chief apothecary, and some others, notwithstanding all their efforts, came to the conclusion that, by chemical analysis, neither alkali, salt, acid, nor a metal, can be discovered in the "aqua Binelli." Whether hereafter the celebrated Berzelius, and other chemists, to whom some of the water has been sent, will succeed in discovering its composition,† remains to be determined.

I have received, from various places, waters possessing an empyreuma, and corresponding in external appearances with the "aqua Binelli," but which, when tried on animals, proved wholly inefficacious.

With respect to the most essential organic changes observed after the employment of this water, I shall for the present only communicate the appearances presented on two post-mortem examinations. The above-mentioned case of amputation afforded the first opportunity, having died the tenth day after the operation. (This patient entered the hospital suffering under violent hectic fever, caused by phthisis ulcerosa pulmonum, and profuse suppuration

* It is proper to state, however, that we have seen the femoral artery of a dog entirely divided with a sharp bistoury, and very trifling hemorrhage followed, although *no means whatever* were had recourse to, to restrain the hemorrhage. In two days the animal appeared in perfect health and strength.—Editors.

† Berzelius has failed.

from a fistulous opening at the knee-joint: the operation was only performed at the extreme and urgent solicitations of the patient himself.) The second was the carotid artery of a horse, which was cut in the direction of its axis, by Dr. Hertwig, and cured by the "aqua Binelli:" the horse was killed fifteen days after the experiment.

A lithographic drawing is attached to the annual report, representing the stump, with the artery and two veins (femoral and saphena) laid open for some inches of their length. The extremity of the artery is perfectly closed by a thrombus, which is cone-shaped and three inches long. A similar, but much shorter coagulum was found in the femoral vein, its cone, or upper extremity, only reaching the nearest valve; from which point another thrombus arose, many inches in length, having its cone at this valve, and its larger extremity towards the heart. The thrombus formed in the saphena vein was similar to that of the artery in every respect. The parietes of the vessels were neither thickened, swollen, nor, on their external side, enclosed by coagulated lymph, as Perot has observed to be generally the case after the obliteration of arteries by ligature. The internal side of the vessels only differed from the normal state by presenting delicate transverse bands, like folds, which were particularly distinct in the artery, and extended somewhat higher than the upper extremity of the coagulum.

A section of the artery of the horse is also represented, showing a coagulum which completely fills the artery to the same extent as the original longitudinal section, thereby closing the wound, and terminating, cone-shaped, at either extremity, above and below the wound.

We refrain from laying before our readers further information at present upon this subject, until all the analyses undertaken to discover the composition of the "aqua Binelli" shall be completed, and a greater number of comparative clinical observations shall be collected.

---

### MISCELLANEOUS.

*Singular Cases of Hereditary Deformity*; communicated by M. Voisin. —*Gazette Medicale*, No. 119.

Case I. Madame Dartijas has had twelve children, nine boys and three girls, and two miscarriages. One of the boys (Louis) has a supernumerary toe on the left foot, situated between the fourth and fifth metatarsal bones; it has two phalanges, and appears to adhere by ligaments only. Another brother (Pierre) has six toes on each foot. The eldest brother (Jean Baptiste) is married, and has had six children; the last has a supernumerary finger at the extremity of the first metacarpal bone of the left hand. One of the sisters (Marie) had an additional finger on the left hand, but, as it was attached by a very slight pedicle, it was removed when she was an infant. Lastly, a sister of Madame Dartijas married, and had eight children; and one of her sons has also a supernumerary finger on the left hand.

Case II. In a family residing at Limoges, a remarkable peculiarity has continued for five or six generations. The eldest male child *alone* has, upon his forehead, a lock of white hair. In the female children no such appearance has ever existed. This inexplicable hereditary phenomenon occurs in every male branch of the family.

Case III. This case and the last were communicated to M. Voisin by his colleague, M. Chastaing. A lady of his acquaintance had a severe whitlow which produced a deformity of her index finger; and a similar deformity was transmitted to her two children. She had had other children before, but in neither of them did any such peculiarity exist.

However inexplicable the above curious facts may be, we are not at all inclined to doubt their authenticity. Two similar cases of hereditary deformity have fallen under our own observation, which we may briefly relate.

CASE I. A lady and her two daughters are now residing in Upper Berkeley street, Portman square. The youngest daughter has a band of perfectly white hair in front, which looks like a broad white ribbon when laid flat round the head: the rest of her hair is black. This peculiarity has existed in one female of several branches of the family for six or seven generations.

CASE II. About sixteen years ago, Mr. NORTH attended a young female during her confinement, who had been the *chère amie* of a banker well known in London. This gentleman has a supernumerary finger on the left hand, and a supernumerary toe on the right foot. Very fortunately for the lady and the infant, the latter bore upon its left hand and foot very striking proofs of its paternal origin, for it had the additional finger and toe. The gentleman no longer doubted the constancy of his mistress, of which he had before been somewhat suspicious, and the child was well educated, and is now in a respectable business.*

We learn, from the Medical Gazette, that there is now in St. George's Hospital, a patient in whom the patellæ are entirely wanting. The knee looks rather flatter than usual, but no apparent evil results from this anomalous formation, as the man says he can walk many miles a day without difficulty. The peculiarity is hereditary, neither his grandfather nor father having had patellæ; and it also extends to other members of his family.

---

*Proportions of Alcohol obtained by* Mr. BRANDE *from different Spirituous Liquors and Wines.*

| | Proportion of Alcohol per cent. by measure. |
|---|---|
| Scotch Whisky | 54.32 |
| Irish Whisky | 53.90 |
| Rum | 53.68 |
| Brandy | 53.39 |
| Hollands | 51.60 |
| Lissa Wine | 24.41 |
| Port, highest | 25.83 |
| lowest | 19.24 |
| average | 22.97 |
| Madeira, average | 22.27 |
| Cape Madeira | 20.51 |
| Currant Wine | 20.55 |
| Sherry, highest | 19.81 |
| lowest | 18.25 |
| average | 19.17 |
| Teneriffe | 19.79 |
| Claret, highest | 17.11 |
| lowest | 12.91 |
| average | 15.10 |
| Burgundy | 14.57 |
| Champagne, still | 13.80 |
| sparkling | 12.80 |
| Hock | 12.08 |
| Gooseberry Wine | 11.34 |
| Tokay | 9.88 |
| Cider | 7.54 |
| Perry | 7.20 |
| Ale (Burton) | 8.88 |
| (Edinburgh) | 6.20 |
| (Dorchester) | 5.56 |
| London Porter | 4.20 |
| Small Beer | 1.28 |

---

*Atomic Theory.* From the sixth edition of MURRAY's *System of Materia Medica and Pharmacy.*

IT is not attempted here to give any outline of the atomic theory, that important doctrine, the influence of which extends through the whole of chemistry, and regulates all the details regarding the combinations of bodies. It may be merely stated, that the leading principle of this doctrine is, that every substance has a certain combining proportion, in which only it will unite with other bodies. Thus, to take the instances of the three simple substances, *hydrogen*, *carbon*, and *oxygen*, the combining weights of these bodies are as the numbers 1, 6, and 8: that is, hydrogen combines with carbon in the proportion of one part of hydrogen with six parts of carbon; hydrogen combines with oxygen in the proportion of one part of hydrogen to eight parts of oxygen; and carbon

* In the estimation of the public, no doubt whatever would be entertained as to who was the father of the child. Even with *such* evidence, however, physiologists might still be sceptical. Vide MAYO's Physiology, 2d edition, p. 489.—EDITORS.

combines with oxygen in the proportion of six parts of the former to eight parts of the latter; or, in the compound of hydrogen and carbon, there will always be six parts by weight of carbon to one part by weight of hydrogen; in the compound of carbon and oxygen, there are always six parts by weight of the former to eight parts of the latter; and in the compound of oxygen and hydrogen, there are eight parts of oxygen to one part of hydrogen. All simple bodies, in like manner, have certain invariable and peculiar combining weights. The reason of this singular law is supposed to be, that chemical combination happens between the atoms, or ultimate particles of bodies, and that the atoms of each body are of a specific size and weight: that is, recurring to the instances which have been given, an atom of carbon is six times the size and weight of an atom of hydrogen, and an atom of oxygen eight times the size and weight of an atom of hydrogen. It is to illustrate this view that the table of the atomic weights of bodies at the commencement of this work is constructed. The atoms of the different simple bodies, which, either in their simple state or in a state of combination, are employed in medicine, are represented of their specific magnitudes in relation to each other, and as denoted by the attached numbers. By the mere inspection of this table, the composition of any compound used in medicine may be determined: thus, if we wish to know the composition of oxide of iron, we observe that the equivalent of oxygen is 8, and that of iron 28; hence, oxide of iron must consist of eight parts of oxygen and 28 parts of iron; adding these together, the sum 36 becomes the equivalent of oxide of iron, and, in any combination of it with an acid, the proportion of the oxide of iron will be as 36 to the equivalent number of the acid. In this simple manner is the whole system of chemical combinations derived from a few numbers.

---

*Average Mortality of Europeans.*

| | | |
|---|---|---|
| Average mortality in | England and Wales, is.... | 1 in 60 |
| | France ................ | 1 in 40 |
| | Sweden and Holland .... | 1 in 48 |
| | Russia ................ | 1 in 41 |
| | Austria ................ | 1 in 38 |
| | Prussia and Naples ...... | 1 in 35 |
| | Leghorn .............. | 1 in 35 |
| | Switzerland ............ | 1 in 49 |
| | Rome . .............. | 1 in 25 |
| | Palermo .............. | 1 in 31 |
| | Madrid ................ | 1 in 29. |

It is said that, in France, about one half the population live to twenty years, and a third to forty-five; the lowest mortality is at the age of ten, being 1 in 180, while at the age of forty it is 1 in 53; the duration of life at fifty is 23 years, and twelve years longer among the rich than the poor. Since the late peace, the governments of Europe have paid great attention to statistics, and we have now returns from all parts of the continent, by which it appears that the mortality of Great Britain, her cities, and her hospitals, is greatly inferior to that of any other country in the old world, and that this nation is the most healthful of all.—*Med. and Surg. Journal.*

---

DIETETICS.

*Portable Milk.* M. Dirchoff, the Russian chemist, who some time since discovered the process of making starch into sugar, has lately made several experiments upon milk: the result which he has arrived at is curious. He is said to have found a mode of keeping milk for use for any definite space of time. The process of preserving is this: he causes new milk to be evaporated over a slow fire, until it is reduced to a powder. This powder is then put into a bottle, which is hermetically sealed. When the milk is wanted for use, it is only to dissolve some of the powder in a seasonable quantity of water, and the mixture so dissolved will have all the qualities, as well as the taste, of milk. —*Edinburgh Agricultural Journal.*

### ZOOLOGY.

*Marvellous Multiplication of the Infusoria.* I have also made some observations on the development and multiplication of the Infusoria, which I deem among the most important of all my researches. I have observed for eighteen days successively a single Hydatina senta, and as it was perfectly grown when I singled it out, and did not die of old age, being accidentally destroyed, the life of this animal must be more than twenty days. Such an individual, when circumstances are favorable, is capable of a fourfold propagation every twenty-four—thirty hours. It can in this time bring forwards four eggs from the embryo state to the exclusion of the young. But this fourfold increase in the space of a day, when no obstacle intervenes, and the same individual gives, in ten days, forty eggs, and raised to the tenth power (therefore on the tenth day) a million of individuals from one mother; and on the eleventh day four, on the twelfth sixteen, millions, &c. Although this productive power is the greatest which has been yet observed in nature, far exceeding that of insects, it is far from attaining that of the Polygastrica. In the Paramœcium aurelia, which is $\frac{1}{12}'''$ in size, and which has been ascertained to live several days, a doubling of each individual by transverse division has been observed within twenty-four hours, its rate of increase is therefore double that of the preceding. But as these animals, besides division, also propagate by eggs, and these eggs are not separated from the parent singly but in masses, and as they also form gems, the possible increase within forty-eight hours becomes quite innumerable. Who can wonder that, under such circumstances, fluids should, with the brood of two or three days only, swarm with these animalcules?—*Edinburgh New Philosophical Journal; from a Paper by* Prof. C. G. Ehrenberg, *of Berlin.*

### FOSSIL BOTANY.

*On the Fossil Flora.* The fact that we do not know more than a few hundred species of fossil plants, shows the impossibility of our pretending at present to form any very plausible estimate or conception of the nature of the antediluvian flora, and also the hazard of attempting, from the disappearance of a few species, in proceeding from one formation to another, to determine the abundance or paucity of families or classes of plants. It is surprising to observe naturalists characterising botanically whole antediluvian epochs, when they possess, as in the case, for example, of the second epoch, the period of formation of the new red sandstone, a group of twenty miserable remains, as the whole of the disposable riches from which such a result is obtained. It is evident also that we cannot, from the present fossil vegetable remains of single families, form any satisfactory inference as to the number of their species, because it may have depended in part on accident, or some unknown circumstances, that in one family many species and individuals are preserved, while in another but few remains are met with. In other cases the circumstances, although easily understood, are not attended to, and the number of fossil species found in the deposit are considered as the full measure of what has been destroyed. Thus, in the first epoch, two mosses only, but sixty-four lycopodiæ, are enumerated; but this circumstance does not prove that mosses and lycopodiæ formerly existed in the proportion of two to sixty-four, for it is very natural that gigantic lycopodium stems would more easily resist the storms of time than the dwarf moss; and although we, on that account, have only two mosses remaining from that period, it does not follow that they have not been more than ten times more numerous than the lycopodia.

It is doubtful if botanists can determine with accuracy even the family of the fossil species. Many of the remains at present considered as Lycopodiums, may prove to be Ferns, or even Pines, the Selaginites excepted. Many of the gigantic, so called Equiseta, resemble tree-like grasses. The Marsileaceæ of the former world may have belonged to the ferns. The Voltzia has not yet been proved to belong to the Coniferæ; and as the confirmation of the coniferous character will give a decided preponderance to this class of plants, in the period in which it occurs, we see from this example how uncertain single conclusions are, which have been considered as sufficient in regard to the prevailing character of many vegetable epochs. An error, which can be so easily committed, in endeavouring to determine fragments very difficult of determination, is doubly prejudicial, when we attempt to found on it a history of the gradual succession of the families of plants. The whole study of the ancient flora, the flora of an early world, is based on such a system of the history of vegetation, and on the proofs that it rises gradually from below upwards in regular successive epochs of more and more perfect vegetables. But in order to judge of this history of vegetation, it is premised that we have previously ascertained the true succession, the internal gradation of vegetation, in short, the true natural vegetable system. But who has discovered this system, and what are its characters?—*Edinburgh New Philosophical Journal.*

